# Supplementary material for: Unequal by the gun: Four decades of the Black-White firearm homicide gap
Source: PLoS One. 2026 Feb 25;21(2):e0341645. doi: 10.1371/journal.pone.0341645 (PMC12935247; doi:10.1371/journal.pone.0341645)
Supplement: Attachment — Submitted filename: racial_gap_in_shootings_rev1_response.pdf [file pone.0341645.s001.pdf]

Dear Editor Dávila-Cervantes,

Thank you for allowing us to revise our manuscript and respond to concerns raised in the reviewers. We have made a number of changes in response to their concerns. Below we provide a point-by-point response to each of the main comments reviewers raised. We are thankful for the constructive reviews, as addressing these comments helped us improve the paper.

Reviewer 1

1.1 The abstract leaves a lot to be desired:

- missing "Background" "Methods" "Results" and "Conclusion" sections
- The data are national for the U.S.
- that there are two calendar-year inflections in increases in the White:Black comparison ("mid-1980s" and 2014).
- that several years had greatest Black vs. White disparity (2020, 2021 and 2023).
- that 31,202 homicides cited as the disparity without indicating over what years and geography (instead expressing the difference with age-adjusted rates).
- Black men were 10.38 times more likely than White men to die from firearm homicide" can be interpreted to mean that a homicide attempt was 10x more likely to result in death.
- "The firearm homicide rate in males was 10 times higher in Blacks than Whites" would be more accurate. [a 2 decimal number is not necessary].
- The "number of excess Black firearm homicide deaths" should be "rate of excess ..." and "homicide deaths" should be "homicides."

Thank you for these comments. We have revised the abstract to address these. The revised version is below:

Background: Firearm homicide is a leading cause of death in the United States, with substantial racial disparities documented over time. Black Americans experience disproportionately higher rates of firearm homicide compared to White Americans, reflecting long-standing social, economic, and structural inequities. This study contributes to understanding the magnitude and trends of this disparity.

Objective: We investigate the Black-White population-level disparity in firearm homicide rates over 45 years in the United States and estimate the fraction of excess deaths in the Black population attributable to firearm homicides.

Methods: We measure racial disparities in firearm homicides as the ratio of age-adjusted rates between Black and White populations. Using annual national firearm homicide and population data from CDC WONDER (1979–2023), disaggregated by race, age, and sex, we calculate age-adjusted Black–White mortality ratios by year and sex, as well as the number of excess Black firearm homicide deaths attributable to these disparities.

Results: After a steady period of 1990-2010, the Black-White disparity began increasing and peaked in 2020 when the firearm homicide rate in males was 10.38 times higher in Blacks than Whites. There were 31,202 excess firearm homicides of Black people attributed to the Black-White racial disparity in 2020-2023.

Conclusions: Despite significant declines in firearm homicides during the 1990s, the relative racial gap in firearm homicide has persisted and grown, reflecting the unequal burden of violence borne by the Black population during both declines and surges of violence.

1.2 Discussion: In general, adequately presented. Not included as potential causes are firearm theft rates differences, varying state regulations in firearm purchasing and ownership, firearm industry policies regarding sales racial pitches, and possibly mass shooting differences in Blacks and Whites.

Thank you for this suggestion. While these sound reasonable, we are not aware of the related literature that would tie these racial disparities with firearm homicides. Additionally, the point of the paper was to point out the massive disparities and how they have changed over time, not to attribute causes. State regulations and mass shootings did not change sufficiently to explain the surges and declines over the past four decades.

1.3 In general, "women" should be "females" and "men" (used 19 times) should be "males."

Thank you, we replaced "men" and "women" with "males" and "females".

1.4 “100 thousand” should be “100,000”

Thank you, we have changed it everywhere accordingly.

1.5 What is meant by “credit” and “other institutions” among the racial inequalities?

Thank you for opportunity to make this more clear. We meant that racially disparate access to various institutions such as finance (loans), healthcare, housing, education forms the backbone of the Black-White racial inequality in the U.S. We changed "credit" to "finance". We also revised the sentence:

These concentrated violence patterns are closely related to long-standing racial inequalities in access to housing, education, finance, criminal justice, health care, employment, and other institutions.

1.6 What does “top 10% of block groups” mean?

Thank you. We meant top 10% of city census block groups ranked by the number of shooting incidents. We revised the sentence accordingly:

For example, in cities such as Philadelphia, New York, and Los Angeles, between 36% and 55% of the increase in shootings between 2020 and 2021 occurred only in the 10% of city census block groups - areas where the residents are overwhelmingly Black and Hispanic.

1.7 How does the reader compare the “greater than 5%” gun injury or death rate in Chicago and Philadelphia with other cities and rural settings if comparative values are not provided?

Thank you. Unfortunately, we do not know of studies that would provide more values for comparison. Instead, we added the comparison made by the study we referred to. It compares 5% annual firearm victimization risk faced by the youth in the most disadvantages areas of Philadelphia and Chicago with wartime mortality and injury rates in the recent US campaigns in Afghanistan and Iraq. A firearm victimization rate of 1,000 per 100,000 population would be a 1% annual risk of firearm victimization. The referenced rate is equivalent to 5,000 per 100,000.

In the most socially disadvantaged neighborhoods of cities like Chicago and Philadelphia, young males face an annual risk of gun injury or death greater than 5%, comparable to or exceeding combat risks of the US service members deployed to Afghanistan and Iraq.

1.8 “in the decade prior” dangles without specifying the actual years

Thank you, added specific years:

Between 2000 and 2010...

1.9 How do “shifts in the age composition” explain the life-expectancy gap between Whites and Blacks?

Thank you. We added a clarification, highlighting that the differences in the age compositions affect the mortality structure:

Between 2000 and 2010, the Black–White life expectancy gap had been narrowing, largely due to shifts in the age composition of these populations, which lead to reductions in mortality differences from chronic diseases.

1.10 “In 2016, homicide mortality for Black males was 20 times higher than for White males .” In the U.S.?

Thank you, we added U.S. to the sentence:

In 2016, homicide mortality for Black males was 20 times higher than for White males in the U.S.

1.11 “‘Firearms are the most significant contributor to homicides among youth and young adults in the U.S., with firearms accounting for approximately 80.5% of all homicides.” 80.5% is quite finite and not “approximate”.

Thank you, corrected:

Firearms are the most significant contributor to homicides among youth and young adults in the U.S., accounting for 81% of all cases

1.12 . . . we follow a similar design and examine the Black-White ratio of firearm homicide mortality rates between 1979 and 2023 after adjusting for sex, age, and year effects.” Since age is in

years, "year" should probably be "calendar year". Other uses of "year' in rest of manuscript should probably specific "calendar year".

Thank you. We revised the sentence to clarify this:

In this paper, we follow the same approach and examine the Black-White ratio of firearm homicide mortality rates between 1979 and 2023 after adjusting for sex, age, and calendar year effects (hereafter, we use "years" to refer to calendar years for brevity)

1.13 Table 1 title/legend lacks description of "Counterfactual".

Thank you, we have added the following description under the table:

Columns "Actual" show counts of Black firearm homicides by age groups and year periods. Columns "Counterfactual" show estimated counts of Black firearm homicides if they had experienced White firearm homicide rates for respective age group and year period. Columns "Excess" show the difference between actual and counterfactual counts.

1.14 Line 172: "similar' would be more appropriate than "same".

Thank you, corrected:

The burden of firearm homicides is disproportionately concentrated among Black males, with Black females having a rate of firearm homicides similar to that of White males.

1.15 Lines 173-174: "Excluding the period of 1989-1993, the decrease in firearm homicide rates for Black males is less pronounced than for other groups" is a qualitive statement that is difficult to understand. If anything, the changes after 2000 suggest that Black and White males had similar trends.

Thank you. We agree and decided to drop this statement.

1.16 Line 207: delete "there'.

Thank you, deleted.

1.17 Table 1: From 1991-1993 to 2018-2020, the excess number of firearm homicides in Blacks decreased in 10-23 year-old, with a total decrease in excess deaths of 2,901. That young Blacks were spared the overall increase from 1991-1993 to 2018-2020 should be recognized and potentially discussed, albeit it is <10% of the overall excess (of all ages).

Thank you for this suggestion. We have added this to the subsection on Excess Deaths:

As can be seen from Table 1, excess firearm homicides of Black youth (age 10-24) are roughly similar across cohorts of 1991-1993 and 2021-2023. The increase in excess Black firearm homicides in 2021-2023 compared to 1991-1993 can be attributed to older age groups of 25 years and older.

1.18 Figure 3: Ideally the White and Black data should be shown on the same graph and given the overlap would only be for 2021-2022 for the single race data of ages 15-19 and 20-24, this option is feasible.

Thank you. We have tried different ways to plot these data, but could not find a better way to present this. As the points plotted on Figure 3 are ratios, disaggregation it into underlying Black and White rates would clutter our presentation. Additionally, firearm homicide rates by race are also presented in Figure 1.

1.19 Figure 4: Does not specify age range.

Thank you. Figure 4 shows total counts of excess Black firearm homicides by each calendar year, and spans all age groups. We have added a short clarification to the Y axis. It now reads:

Excess Black deaths from firearm homicides (all ages).

1.20 Acknowledgements: Why does only one of the two authors acknowledge the source of financial support?

Thank you. One of the authors included this by mistake, and in the revised submission, no longer acknowledges financial support.

1.21 References: Their formatting and content is inconsistent, with doi in some an not others and some titles capitalized and in others not. Internet citations lack date of access. #13 had two doi citations. #9 and #18 repeats the e number. #23, #24, #37 and #40 appear to be incomplete.

Thank you. We have revised our bibliography: unified DOI links, added date of access for Internet sources, and corrected the above mentioned bibliography items.

Reviewer #2

2.1 By my calculation, the total # of excess deaths in the thee key peak periods that the authors look at is 81,291. I think it may be helpful for the authors to contextualize this. One way to do this is the following. The Dallas Cowboys NFL Stadium holds around 80,000 people (can be expanded for standing room only). Taking the # of excess deaths means that all those deceased persons can fit in the stadium. There likely is a better way to say this but hopefully the authors get the point. That kind of visual, however depressing, is powerful.

Thank you. This is a great idea. We have not found a way to incorporate into the manuscript nicely. However, this would be a great thing to note in a press release or op-ed.

2.2 I know the authors did not do anything w/ Hispanic ethnicity (though they could look at it in more recent times). Could they do a subset analysis of Hispanic ethnicity (I am not requiring it), but if not at least comment on it.

Thank you. This is an excellent suggestion. Hispanic ethnicity is completely separate from racial classification in CDC WONDER data. Doing this follow-up analysis requires to fully revise data preparation, and we are afraid this will be beyond current paper, which focuses on Black-White racial gap.

2.3 The paper lends heavily on structural explanations of the differential risk of firearm victimization. I do not discount that this matters, however, people still have to pick up guns AND use them. There still remains some sort of individual-level decision making here, whether its code of the street, self-control, etc. I think the authors need to point that out.

We tried to keep the language to noting larger inequalities in society. We agree with the reviewer, but we don't want this paper to get misconstrued into a causal explanation for the disparities.

2.4 I like descriptive papers. A lot. Yet, not much policy commentary is here. I think a sentence or two would be useful.

We added a few sentences on policy evaluation designs that would be needed:

...research is needed to understand how local contexts and policies are associated with the racial gap in firearms homicide. Policy evaluations and quasi-experimental studies are required to identify which neighborhood-level characteristics or interventions are effective at reducing these disparities over time.

Reviewer #3

3.1 However, I am concerned by the absence of a substantive investigation into racism as a driver of these trends. The framing largely gives a cursory mention of socioeconomic and neighborhood-level characteristics without adequately addressing the structural and systemic racism that underlies and shapes these factors. Without explicitly engaging with racism as a root cause, the analysis risks reinforcing race as a proxy for other factors rather than interrogating the historical and ongoing policies, practices, and power structures that produce and maintain these disparities. Integrating a discussion of racism—at minimum through a theoretical lens, but ideally via an empirical consideration—would strengthen the manuscript, situating the observed trends within the broader social and political context that creates disparities in firearm homicide.

Thank you. The introduction reviews the existing evidence about racial disparities in homicides and refers to some of the literature that mentions structural racism in the U.S. We think highlighting this inequality gets to the concerns regarding the structural sources that drive this enduring Black-White gap in homicide.

3.2 Introduction: This section is a bit lengthy and redundant in places. For example, the authors include a lengthy discussion the place-boundedness of these racial disparities, but since this is not the focus of the paper, this section could be reduced or moved to the discussion.

Thank you. The point about concentration of violence in Black and Hispanic neighborhoods literally only takes first two sentences in the Intro. The remaining seven paragraphs of the Introduction review existing evidence on racial disparities in the U.S., particularly in homicide and violence. We have edited it again to try and reduce redundancies.

3.3 The introduction is missing several references to existing literature on firearm homicide disparities. These may also fit in the discussion.

a. Wong B, Bernstein S, Jay J, Siegel M. Differences in racial disparities in firearm homicide across cities: the role of racial residential segregation and gaps in structural disadvantage. Journal of the National Medical Association. 2020 Oct 1;112(5):518-30.

b. Conrick KM, Adhia A, Ellyson A, Haviland MJ, Lyons VH, Mills B, Rowhani-Rahbar A. Race, structural racism and racial disparities in firearm homicide victimisation. Injury prevention. 2023 Aug 1;29(4):290-5.

c. Siegel M, Rieders M, Rieders H, Moumneh J, Asfour J, Oh J, Oh S. Measuring structural racism and its association with racial disparities in firearm homicide. Journal of racial and ethnic health disparities. 2023 Dec;10(6):3115-30.

d. Knopov A, Rothman EF, Cronin SW, Franklin L, Cansever A, Potter F, Mesic A, Sharma A, Xuan Z, Siegel M, Hemenway D. The role of racial residential segregation in Black-White disparities in firearm homicide at the state level in the United States, 1991-2015. Journal of the National Medical Association. 2019 Feb 1;111(1):62-75.

e. Bottiani JH, Camacho DA, Lindstrom Johnson S, Bradshaw CP. Annual research review: youth firearm violence disparities in the United States and implications for prevention. Journal of child psychology and psychiatry. 2021 May;62(5):563-79.

Thank you, these references are relevant. We have reviewed them, and added some of them to the introduction:

Several studies have analyzed racial disparity in firearm homicides at sub-national level in the United States and found robust evidence of the related Black-White gap [31-33].

3.4 Methods: Why did the authors not conduct formal trend analyses, such as via Joinpoint? This would allow the conclusions regarding spikes to be more sound.

Thank you for this suggestion. Conducting formal trend analysis is beyond the scope of the paper. Instead, we focus on calculating annual age-adjusted measure of racial gap over 40 years, and estimated the related excess deaths.

3.5 Results: The terminology of "fatal shootings" is new in this section and could be misleading. Please specify the unit is firearm homicides per 100 thousand people. Additionally, were these truly whole numbers? That is "7.0 per 100 thousand;" if not, please include the precise numbers.

Thank you, this is very helpful. We have revised the numbers and the wording:

The highest rate of 7.20 firearm homicides per 100,000 people occurred in 1993, while there were a total of 6.36 firearm homicides per 100,000 in 2020.

3.6 Please be consistent in using terms related to sex (male, female) compared to gender (men, women).

Thank you, we have corrected it throughout the manuscript.

3.7 It would be helpful to discuss in what ways trends changed with the change in race definitions.

Thank you. We think it's too early to discuss this change: there are only three years of data post-change. Besides, the change itself does not affect our results but increases the uncertainty of the estimated rates. We have briefly reviewed this in the Data section:

To address the change in data categorizations, we proceed with our analysis by combining UCD's four bridged-race categories data in 2018-2020 and six single-race categories data in 2018-2023. Single-race categories are created in two possible variations: with and without adding "More than one race" records to Black and White population and death counts. While this does not allow us to perfectly align post-2020 estimates with previous years, it creates upper and lower bounds for the change in 2021-2023, allowing us to see the direction of change in the racial gap in homicide mortality. Importantly, the change in CDC's race and ethnicity categories does

not affect the estimates of excess deaths, as can be seen from Figure 4.

3.8 In Figure 1, the difference in the dashed line between single race and single + multi race is difficult to distinguish. Consider making one a dotted line. Additionally, the major differences in y-axes make it difficult to make visual comparisons.

Thank you. We have changed the line types to solid, dotted, and dashed, which improved readability. We deliberately decided to make y-axis flexible to show the similar trends among Black and White males and females as opposed to their baseline firearm homicide rates.

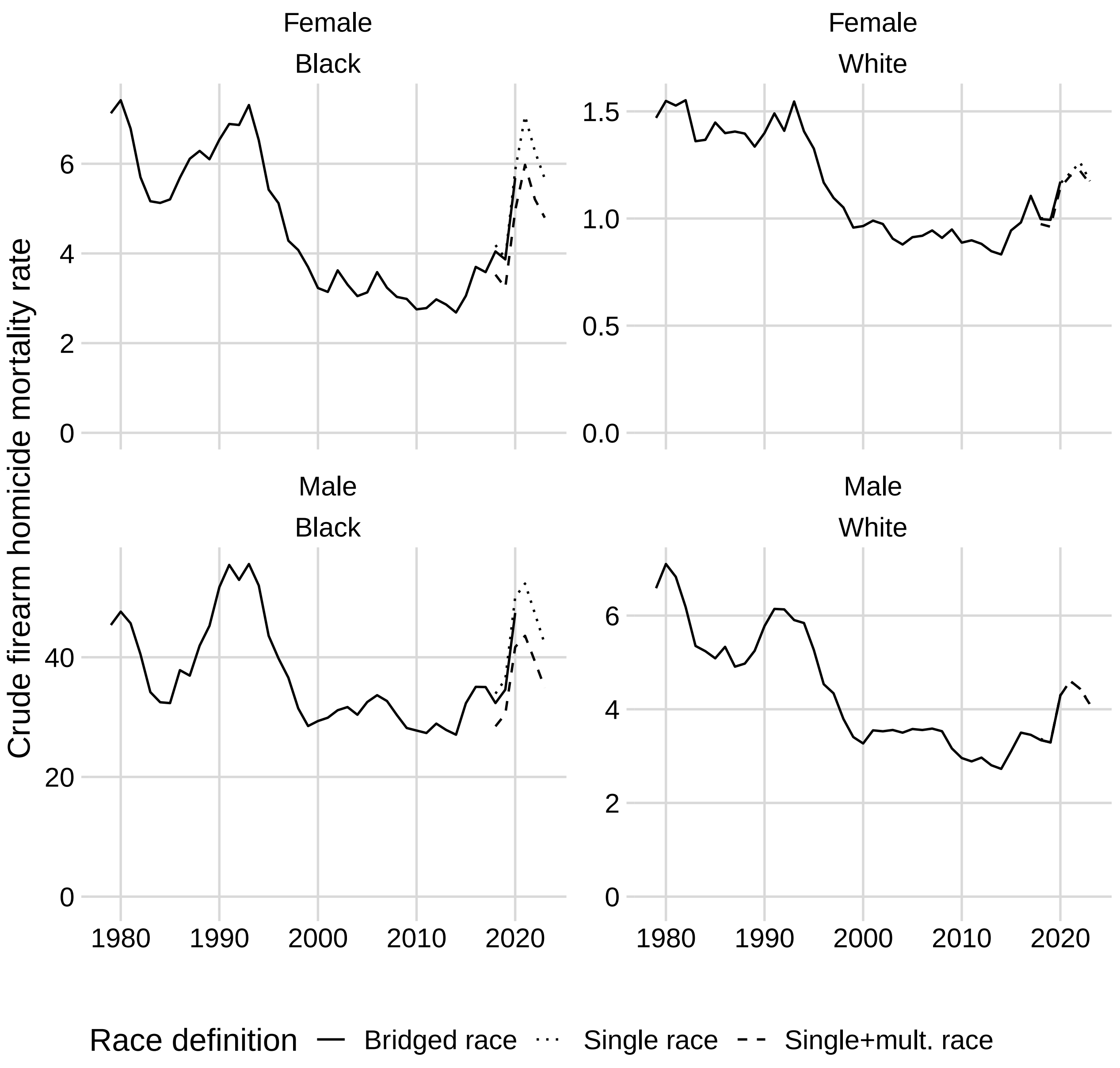

Figure 1: **Crude firearm homicide rates in the United States in 1979-2023.** Rates are not adjusted for age and represent counts of firearm homicides per 100,000 population.

3.9 In Figures 1-2, what is the unit for the y-axis? Per 100 thousand?

The unit of analysis in Figure 1 is crude firearm homicide rate per 100,000. The unit of analysis in Figure 2 is mortality ratio of Black firearm homicide rate to White homicide rate. We have added these explanations into respective Figure captions.

3.10 The discussion needs expanded explanations for these trends. Remarkably, the word "racism" does not appear at all in the manuscript. Please see: `https://www.healthaffairs.org/content/`

`forefront/racism-new-standard-publishing-racial-health-inequities`

Thank you for this comment. We have added a sentence to the Discussion section that cites studies which invoke structural racism as a factor contributing to racial disparities in firearm victimization, along with several supporting references. However, we have chosen not to use the term "racism" explicitly, as we believe the evidence does not allow us to definitively establish racism as the causal factor.

The long-standing racial disparities in violent and firearm victimization in the U.S. are tied to other dimensions of structural inequality [3-7].

3.11 "At the same time, there is no clear causal evidence for the rise in homicide and the growth in the Black-White inequality in firearm homicides that started in 2015 and accelerated during the COVID-19 pandemic." This sentence is a bit misleading; I believe the authors mean to say that this literature is nascent, and we are still identifying the causal pathway. However, it may be misinterpreted to mean that there is no causal relationship at all, rather than that the current evidence base is limited and evolving. Clarifying that the body of research is emerging, and that causal mechanisms are still being explored, would help avoid confusion.

Thank you. We have added this clarification to the sentence as follows:

At the same time, there is no clear evidence for causal pathways between the rise in homicide and the growth in the Black-White inequality in firearm homicides that started in 2015 and accelerated during the COVID-19 pandemic.

3.12 Regarding the section on poverty, please also see:

a. Ellyson AM, Rivara FP, Rowhani-Rahbar A. Poverty and firearm-related deaths among US youth. JAMA pediatrics. 2022 Feb 1;176(2):e214819-.

b. Barrett JT, Lee LK, Monuteaux MC, Farrell CA, Hoffmann JA, Fleegler EW. Association of county-level poverty and inequities with firearm-related mortality in US youth. JAMA pediatrics. 2022 Feb 1;176(2):e214822-.

Thank you. We have reviewed these papers and cited one of them in the Introduction.

3.13 Please remove the recommendation that future studies control for neighborhood socioeconomic characteristics for two reasons. First, an extensive body of literature already examines neighborhood characteristics and disparities in firearm homicide. Second, rather than controlling for these characteristics, it would be more useful for future research to identify which specific characteristics are associated with higher or lower disparities.

Thank you. We have revised this part:

Second, given that the Black population in the U.S. is experiencing disproportionately more concentrated disadvantage, further studies might look at the long-term trends of firearm homicides while simultaneously controlling for socioeconomic characteristics of neighborhoods. An extensive literature already documents these trends at the level of states, counties, and selected cities [32-35]. More research is needed to understand how local contexts and policies are associated with the racial gap in firearms homicide. Policy evaluations and quasi-experimental studies are required to identify which interventions are effective at reducing these disparities over time.
