# Supplementary material for: Unequal by the gun: Four decades of the Black-White firearm homicide gap
Source: PLoS One. 2026 Feb 25;21(2):e0341645. doi: 10.1371/journal.pone.0341645 (PMC12935247; doi:10.1371/journal.pone.0341645)
Supplement: Attachment — Submitted filename: racial_gap_response_v2.pdf [file pone.0341645.s002.pdf]

Dear Editor Davila-Cervantes,

Thank you for the opportunity to address the remaining comment from Reviewer #3. The reviewer notes that "adding a sentence citing literature that 'invokes' structural racism but declining to use the term 'racism' explicitly because of concerns about causal certainty... does not address the core issue," suggesting that our treatment of the topic is cursory. We respectfully disagree. Highlighting large population-level disparities in firearm victimization that have grown in recent years directly engages a critical public health concern disproportionately affecting African American communities.

Reviewer #3's comment appears to reflect an editorial rather than an empirical concern. Our initial decision not to use the term "racism" explicitly was based on its lack of direct measurability in our analysis. For example, racism does not necessarily vary in a way that would explain year-to-year fluctuations in firearm homicides. Nonetheless, to respond substantively to the reviewer's concern, we have revised the text in both the Introduction and Discussion to explicitly incorporate the term "racism" within the appropriate conceptual context, noting that long-standing racial disparities in housing, poverty, and disinvestment are endemic and may reflect structural racism.

Specifically, in the Introduction we changed:

"Scholars identify these key structural forces as the main drivers of racial gaps in mortality and life expectancy in the United States."

to:

"Scholars identify these key structural forces as the main drivers of racial gaps in mortality and life expectancy in the United States, noting that they reflect evidence of structural racism."

In the Discussion we changed:

"The long-standing racial disparities in violent and firearm victimization in the U.S. are tied to other dimensions of structural inequality."

to:

"The long-standing racial disparities in violent and firearm victimization in the U.S. are tied to other dimensions of structural inequality, and suggest action is needed to address these enduring differences by race."

We have also added a citation that directly addresses structural racism in the context of racial inequities in firearm violence: Jay J. Structural Racism and Long-term Disparities in Youth Exposure to Firearm Violence. JAMA Netw Open. 2023;6(5):e2312425. doi:10.1001/jamanetworkopen.2023.12425

We hope these revisions address Reviewer #3's concerns. We also note that PLOS ONE's publication criteria do not require the use of any particular theoretical language. Rather, the journal specifies that accepted manuscripts must: (1) present original, unpublished research; (2) employ rigorous methodology with transparent, reproducible reporting; (3) draw conclusions supported by the data; (4) be written clearly in standard English; (5) comply with ethics and research-integrity standards; (6) adhere to appropriate reporting guidelines; and (7) make data (and code, where applicable) openly available. We believe our manuscript meets all of these criteria. While we have incorporated the reviewer's suggestions in good faith, we do not believe the paper should be required to adopt a specific theoretical framing or function as an editorial on structural racism, particularly given PLOS ONE's stated policy that it does not publish editorial-style articles.

Additionally, we have finalized the publicly available replication package containing code and data. Its blinded version is available here: `https://anonymous.4open.science/r/racial_gap_gun_homicides/README.md`. We added a sentence to the first paragraph in Materials and Methods section:

...We did not have access to personally identifiable information and compiled these publicly available datasets in aggregated form on December 1, 2022 and then accessed new years of data on June 15, 2025. The replication package containing data and code is available here: `https://anonymous.4open.science/r/racial_gap_gun_homicides/README.md`.

When/if the manuscript is accepted, we will replace the blinded link with a direct link to a public Github repository.
